# Contribution of Total Screen/Online-Course Time to Asthenopia in Children During COVID-19 Pandemic *via* Influencing Psychological Stress

**DOI:** 10.3389/fpubh.2021.736617

**Published:** 2021-12-01

**Authors:** Lin Li, Jing Zhang, Moxin Chen, Xue Li, Qiao Chu, Run Jiang, Zhihao Liu, Lili Zhang, Jun Shi, Yi Wang, Weizhong Zhu, Jian Chen, Pengcheng Xun, Jibo Zhou

**Affiliations:** ^1^Department of Ophthalmology, Shanghai Ninth People's Hospital, School of Medicine, Shanghai Jiao Tong University, Shanghai, China; ^2^Center for Single-Cell Omics, School of Public Health, School of Medicine, Shanghai Jiao Tong University, Shanghai, China; ^3^China Hospital Development Institute, School of Medicine, Shanghai Jiao Tong University, Shanghai, China; ^4^Dongtai Tangyang High School, Yancheng, China; ^5^Department of Ophthalmology, Jiaxing First Hospital, Jiaxing, China; ^6^Department of Ophthalmology, Shouxian Hospital, Huainan, China; ^7^Huangpu District Center for Disease Control and Prevention, Shanghai, China; ^8^Department of Epidemiology and Biostatistics, School of Public Health-Bloomington, Indiana University, Bloomington, IN, United States; ^9^Department of Global Value Access and Outcomes, Atara Biotherapeutics, Thousand Oaks, CA, United States

**Keywords:** asthenopia, school-aged children, online-course time, psychological stress, COVID-19

## Abstract

**Objectives:** During the coronavirus disease 2019 (COVID-19) self-quarantine period, the transition to online-course has profoundly changed the learning modes of millions of school-aged children and put them at an increased risk of asthenopia. Therefore, we aimed to determine associations of the total screen/online-course time with asthenopia prevalence among that children during the COVID-19 pandemic, and whether the associations were mediated by psychological stress.

**Methods:** Asthenopia was defined according to a validated computer vision syndrome questionnaire (CVS-Q). We used CVS-Q to collect the frequency and intensity of 16 asthenopia-related eye symptoms of 25,781 children. Demographic features, eye care habits, visual disorders, lifestyle, psychological and environmental factors, were also collected.

**Results:** The overall asthenopia prevalence was 12.1%, varying from 5.4 to 18.2% across grade/gender-classified subgroups. A 100-h increment of total screen/online-course time were associated with an increased risk of asthenopia by 9% [odds ratio (OR) = 1.09] and 11% (OR = 1.11), respectively. Mediation analysis showed that the proportions of total effects mediated by psychological stress were 23.5 and 38.1%, respectively. Age, female gender, having myopia or astigmatism, bad habits when watching screens were also risk factors. Conversely, keeping 34–65 cm between eyes and screen, increased rest time between classes, and increased eye exercise were all associated with a decreased risk.

**Conclusion:** Our study indicated that the influence of long total screen or online-course time on psychological stress increases asthenopia risk. The findings of this study have provided a new avenue for intervening screen-related asthenopia in addition to incorporating a reasonable schedule of online courses into educational policy.

## Introduction

In late December 2019, Wuhan City, Hubei Province, China reported patients with viral pneumonia caused by a new virus, later named “severe acute respiratory syndrome coronavirus 2 (SARS-CoV-2)” (1). Shortly after that, evidence of person-to-person transmission of SARS-CoV-2 was reported (2). This infection was implicated in the onset of severe respiratory illness clusters associated with intensive care unit admission and high mortality, which was called “2019 novel coronavirus disease (COVID-19)” (3, 4). The “WHO COVID-19 Weekly Epidemiological Update” online stated that as of June 27, 2021, more than 180 million cases had been confirmed worldwide with a fatality rate of about 2.2% (WHO, 2021) (5).

In response to the COVID-19 pandemic, Chinese schools were shuttered to prevent the spread of infection. Public events such as meetings, festivals, sporting events, and religious activities were also discouraged. The Ministry of Education estimated that more than 220 million children and adolescents were confined to their homes (6). During the pandemic, significant efforts have been made to create online courses at different levels (6), which were offered as early as mid-February that was 3 weeks after the Chinese Spring Festival. Although online courses are necessary, they require many efforts to focus on various objects at different distances from the eyes. The more attempts the eyes make to focus clearly on the objectives, the more pressure is placed on the intraocular muscles, producing eye strain and headaches. Therefore, asthenopia, mainly characterized by eye strain, eye pain, dry eyes, itching eyes, and headaches (7), often occurs after heavy use of digital devices and excessive screen time exposure.

Asthenopia prevalence among school-aged children and adolescents varies significantly across countries, ranging from 12.4 to 57.9% in studies conducted in India (8, 9), Brazil (10), Italy (11), England (12), Australia (13), and Sweden (14, 15). Several studies have shown that increased use of digital tools is positively related to asthenopia risk among college students and school-aged children (12, 16).

However, direct epidemiological evidence on asthenopia prevalence and its related risk factors among Chinese children during the COVID-19 self-quarantine period is lacking. Importantly, prolonged school closure and home confinement may have added additional psychological stress to children (17). Therefore, in this study, we aimed to examine (1) the associations of the total screen or online-course time with asthenopia risk, and (2) whether psychological stress mediates the associations, using data from a cross-sectional survey conducted among school-aged children in eastern China.

## Materials and Methods

### Study Design, Setting, and Participants

We used a three-stage sampling strategy to conduct this cross-sectional survey of 8–20 years old students. First, we selected five provinces/municipalities, including Shanghai, Jiangsu, Zhejiang, Shandong, and Anhui, in the Huadong region (Eastern China). Second, in each province (or municipality), we selected one to two prefecture-level cities. Third, we selected an average of two elementary schools and three middle/high schools in each city. Finally, we got a sample of 25,781 students from 13 elementary schools, 11 middle schools, and 11 high schools.

The questionnaire consisted of the online version of a validated computer vision syndrome questionnaire (CVS-Q) (18), Chinese eye health and behavior questions (19), and questions about lifestyle, demographic factors, and psychological stress. We completed the online questionnaires between March 28 and May 1, 2020, during the COVID-19 pandemic self-quarantine period. This study protocol was reviewed and approved by the Ethics Committee of Shanghai Ninth Hospital, School of Medicine, Shanghai Jiao Tong University (SH9H-2020-T304-1). Electronic informed consent was obtained from each participant.

Among 25,781 students participated in the online survey, 3,815 (14.8%) were sequentially excluded for without consent (*n* = 150), incomplete information (*n* = 1,130), or unreasonable values of critical variables (*n* = 2,535) (Supplementary Figure 1). The final analysis included the remaining 21,966 children.

### Measurement of Total Screen/Online-Course Time

We considered the total screen time and total online-course time as two major exposures. The total screen time was defined as the time (hours) spent on the screens for online courses, homework, computer games, movies, and TV shows. It was calculated by multiplying daily screen time (hours/day) with the self-quarantined period (days) (20). Similarly, the daily time spent on online courses (hours/day) and the self-quarantined period (days) were multiplied to estimate the total online-course time (hours).

### Measurement of Asthenopia

The CVS-Q for determining visual fatigue (18) was translated into Chinese. Detailed information on items of the questionnaire and the validation were reported in “Supplementary Method 1” and “Supplementary Method 2,” respectively. Briefly, all participants were asked about the frequencies and the intensities of 16 related symptoms. The score of each item was defined as the multiplication of its frequency and intensity; the total score was the sum of all item scores. If the total score was ≥ 6, then the student was considered to suffer asthenopia. The validation was conducted in a pilot study (*n* = 516) using the item response theory (IRT) (21).

### Measurement of Mental Health

We specially designed questions to investigate the perceived stress of students, their sources of stress, and concerns about themselves and the coronavirus infection of their family members. All the items of perceived stress and concerns about one and/or own family being infected by coronavirus were rated on a five-point response scale (0 = never, 1 = almost never, 2 = sometimes, 3 = fairly often, 4 = very often). The total perceived stress score was positively correlated with the severity of perceived stress (22).

### Measurement of Eye Use Behavior Habits and Other Covariates

Questions on eye care behavior habits included wearing glasses, posture during digital device use, light intensity, the distance between eyes and screen, eye rest frequency between classes, eye exercise frequency, and eye drop usage. Other information included basic demographic features (e.g., age, gender, grade, and school), refractive error (e.g., myopia and astigmatism), and lifestyle factors (e.g., physical activity, sleep time, and eating habits).

### Statistical Analysis

All analyses were conducted using STATA 16 (College Station, TX: Stata Corp LLC, USA) (23). A two-sided *P*-value of ≤ 0.05 was considered statistically significant.

Data were presented as means (*SD*s), medians (25th−75th percentiles), or percentages. The differences in characteristics between those with and without asthenopia, or across quartiles of the total screen/online-course time, were compared using Student's *t*-test for independent two samples/ANOVA, Chi-squared test, or Wilcoxon rank-sum test/Kruskal-Wallis equality-of-population rank test, as appropriate. To assess the association between the total screen/online-course time and asthenopia risk, we used mixed-effects logistic regression (24) with individuals (level 1) nested in school (level 2) and province (level 3), reporting odds ratios (ORs) and 95% confidence intervals (Cis). We selected potential confounders among demographic factors, eye health and behavior factors, lifestyle factors, and psychological stress factors associated with both the exposure and the outcome. Stratified analyses were conducted based on gender and graduating status (yes vs. no) [pre-specified], and *p*-values for interaction were obtained by using the log-likelihood ratio test. We tested linearity by using an exposure of interest as a continuous variable. We reported the associations (OR and 95% CI) for per 100-h increment in the exposure and the associations of covariates included in the final models with the outcome.

To examine whether psychological stress mediates the above associations, we conducted a causal mediation analysis using a counterfactual framework-based approach (25). We calculated the direct effect and indirect effect (i.e., mediation effect), as well as the proportion mediated. We used the ordinary logistic regression model in the mediation analysis for simplicity since we did not find a substantial difference between the results from mixed-effects logistic regression and those from the ordinary logistic regression (Supplementary Tables 1, 2).

## Results

### Characteristics of the Study Population by Asthenopia Status

Among 21,966 participants, 2,647 participants (12.1%) were assessed as asthenopia (Table 1). Overall, the students had a mean (*SD*) age of 13.8 [2.4]. Among them, 47.2% were women, and 70% were from cities or counties. 63.1% of the students had myopia, and 36% had astigmatism.

**Table 1:** Characteristics of the study population by asthenopia status^a^.

	**Total**	**Asthenopia**		***P*-Value^b^**
		**No**	**Yes**	
No. of participants (%)	21 966 (100.0)	19 319 (87.9)	2,647 (12.1)	NA
Total screen time (hours)	175.0 (84.0–350.0)	168.0 (77.0–336.0)	294.0 (126.0–490.0)	<0.001
Total online-course time (hours)	140.0 (84.0–280.0)	126.0 (84.0–280.0)	224.0 (126.0–350.0)	<0.001
Study time without a screen (hours/day)	2.7 (2.1)	2.7 (2.1)	2.7 (2.0)	0.253
Rest between classes				
Rest frequency (times/day)	4.0 (1.9)	4.0 (1.9)	4.1 (2.1)	0.027
Rest time (minutes/day)	25.1 (23.2)	25.7 (23.2)	21.3 (22.3)	<0.001
Rest activity, No. (%)				<0.001
Look out of a window	8,388 (38.2)	7,649 (39.6)	739 (27.9)	
Use a cellphone	2,152 (9.8)	1,658 (8.6)	494 (18.7)	
Read books	1,532 (7.0)	1,335 (6.9)	197 (7.4)	
Close eyes	3,777 (17.2)	3,239 (16.8)	538 (20.3)	
Others	6,117 (27.8)	5,438 (28.1)	679 (25.7)	
Demographic factors				
Age (year)	13.8 (2.4)	13.6 (2.3)	15.1 (2.2)	<0.001
Girl, No. (%)	10 361 (47.2)	8,945 (46.3)	1,416 (53.5)	<0.001
Administrative district, No. (%)				<0.001
City	8,819 (40.1)	7,826 (40.5)	993 (37.5)	
County	6,563 (29.9)	5,820 (30.1)	743 (28.1)	
Town	2,827 (12.9)	2,401 (12.4)	426 (16.1)	
Countryside	3,750 (17.1)	3,272 (16.9)	485 (18.3)	
Eye health and behavior				
Myopia, No. (%)	13 868 (63.1)	11 699 (60.6)	2,169 (81.9)	<0.001
Astigmatism, No. (%)	7,904 (36.0)	6,456 (33.4)	1,448 (54.7)	<0.001
Glasses-wearing, No. (%)				<0.001
Never	8,656 (39.4)	8,110 (42.0)	546 (20.6)	
Occasionally	3,675 (16.7)	3,108 (16.1)	567 (21.4)	
Always	9,635 (43.9)	8,101 (41.9)	1,534 (58.0)	
Lying down or lying on the stomach while watching a screen, No. (%)				<0.001
Never	6,826 (31.1)	6,315 (32.7)	511 (19.3)	
Occasionally	12 985 (59.1)	11 366 (58.8)	1,619 (61.2)	
Often	1,934 (8.8)	1,498 (7.8)	436 (16.5)	
Always	221 (1.0)	140 (0.7)	81 (3.1)	
Distance from eyes to the screen, No. (%)				<0.001
≤ 33 cm	2,192 (10.0)	1,972 (10.2)	220 (8.3)	
34–65 cm	3,793 (17.3)	3,420 (17.7)	373 (14.1)	
≥66 cm	15 981 (72.8)	13 927 (72.1)	2,054 (77.6)	
Eye exercise, No. (%)				<0.001
0 times/week	10 059 (45.8)	8,510 (44.1)	1,549 (58.5)	
1–4 times/week	3,546 (16.1)	3,180 (16.5)	366 (13.8)	
5–6 times/week	3,585 (16.3)	3,330 (17.2)	255 (9.6)	
≥7 times/week	4,775 (21.7)	4,298 (22.2)	477 (18.0)	
Eye drops for foreign body sensation,dry or fatigue eyes, No. (%)				<0.001
0 times/day	17 096 (77.9)	15 302 (79.2)	1,794 (67.8)	
1 times/day	1,305 (5.9)	1,096 (5.7)	209 (7.9)	
2 times/day	2,764 (12.6)	2,334 (12.1)	430 (16.2)	
≥3 times/day	795 (3.6)	581 (3.0)	214 (8.1)	
Lifestyle factors				
Physical activity				
Duration (hours/day)	1.1 (0.9)	1.1 (0.9)	1.0 (0.9)	<0.001
Intensity, No. (%)				<0.001
Light-intensity	7,731 (35.2)	6,588 (34.1)	1,143 (43.2)	
Moderate-intensity	13 674 (62.3)	12 277 (63.5)	1,397 (52.8)	
Vigorous-intensity	561 (2.6)	454 (2.4)	107 (4.0)	
Physically active, No. (%)^c^	8,874 (40.4)	8,040 (41.6)	834 (31.5)	<0.001
Sleep time (hours/day)	8.4 (1.2)	8.4 (1.1)	7.8 (1.2)	<0.001
Sleep time, No. (%)				<0.001
<8.0 h/day	4,496 (20.5)	3,467 (17.9)	1,029 (38.9)	
8.0–9.9 h/day	14 107 (64.2)	12 691 (65.7)	1,416 (53.5)	
≥10.0 h/day	3,363 (15.3)	3,161 (16.4)	202 (7.6)	
Change in diet habit, No. (%)	4,091 (18.6)	3,321 (17.2)	770 (29.1)	<0.001
Psychological stress
Perceived stress score	5.4 (2.9)	5.2 (2.8)	7.6 (3.0)	<0.001
Worry about COVID-19	1.6 (1.1)	1.5 (1.1)	1.7 (1.1)	<0.001

*COVID, coronavirus disease; Q, quartile; SD, standard deviation*.

a *Data values were mean (SD), median (25^th^-75^th^ percentile), or number (percentage)*.

b *P-values for the overall difference in related variables across quartiles of the total screen/online-course time were calculated using Student's t-test for independent two samples, chi-squared test, or Wilcoxon rank-sum test, as appropriate*.

c *Participants were considered physically active if they did moderate or vigorous activity ≥ 4 times/week and ≥ 30 min/day*.

As compared to their peers without asthenopia, those students with asthenopia had longer screen/online-course time, more frequent rests, and less daily rest time, and were less likely to look out of the window and more likely to use a cell phone. They were older and more likely to be women, and come from towns or countryside. They had a higher proportion of myopia or astigmatism and were more likely to wear glasses, lie down or lie on their stomachs while looking at the screen. They also tended to keep a longer distance between eyes and screen (≥66 cm), reduce eye exercise frequency, and use eye drops more frequently. Also, they tended to sleep less, had a higher chance of changing their eating habits, had a higher average score of perceived stress, and were more concerned about COVID-19.

### Characteristics of Participants According to Total Screen/Online-Course Time

Compared to those with the lowest quartile of the total screen/online-course time, those in the top quartile rested more frequently but had less rest time. They were less likely to look out of the window and more likely to use a mobile phone (Table 2). They were older, more likely to be women, and come from cities. They were also more likely to suffer from myopia and astigmatism, and have bad habits when watching screens. The possibility of wearing glasses was higher, but the chance of eye care (doing eye exercise and using eye drops ≥ three times/day) was lower. Additionally, they were less physically active, with shorter active hours, and more likely to engage in light-intensity activities. They slept less and had a higher chance of changing their eating habits. Moreover, they had higher perceived stress and fewer concerns about COVID-19.

**Table 2:** Characteristics of the study population by quartiles of total screen time/online-course time^a^, ^b^.

	**Quartiles of total screen time, hours**				**Quartiles of total online-course time, hours**			
	**Q1 (lowest)**	**Q2**	**Q3**	**Q4 (highest)**	**Q1 (lowest)**	**Q2**	**Q3**	**Q4 (highest)**
No. of participants	6,344	4,879	5,442	5,301	6,732	4,468	5,372	5,394
Total screen/online-course time (hours)	67.2 (42.0–67.2)	140.0 (112.0–168.0)	252.0 (224.0–315.0)	504.0 (420.0–616.0)	56.0 (42.0–84.0)	126.0 (112.0–126.0)	210.0 (168.0–252.0)	392.0 (336.0–490.0)
Study time without a screen (hours/day)	2.2 (2.0)	2.9 (2.2)	3.0 (2.1)	2.9 (1.9)	2.5 (2.1)	2.5 (2.0)	2.9 (2.1)	2.9 (2.0)
Rest between classes								
Rest frequency (times/day)	3.8 (1.5)	3.8 (1.7)	4.1 (1.9)	4.6 (2.3)	3.5 (1.5)	4.0 (1.6)	4.1 (1.9)	4.7 (2.3)
Rest time (minutes/day)	29.7 (22.4)	28.1 (26.6)	23.7 (23.2)	18.4 (18.4)	29.4 (25.9)	28.2 (22.2)	23.9 (22.8)	18.6 (18.6)
Rest activity, No. (%)								
Look out of a window	3,078 (48.5)	1,866 (38.2)	1,882 (34.6)	1,562 (29.5)	2,752 (40.9)	1,998 (44.5)	1,819 (33.9)	1,829 (33.9)
Use a cellphone	263 (4.2)	302 (6.2)	613 (11.3)	974 (18.4)	358 (5.3)	279 (6.2)	675 (12.6)	840 (15.6)
Read books	455 (7.2)	369 (7.6)	370 (6.8)	338 (6.4)	508 (7.5)	315 (7.1)	397 (7.4)	312 (5.8)
Close eyes	1,000 (15.8)	747 (15.3)	952 (17.6)	1,071 (20.2)	943 (14.0)	719 (16.1)	987 (18.4)	1,128 (20.9)
Others	1,548 (24.4)	1,595 (32.7)	1,618 (29.7)	1,356 (25.6)	2,171 (32.2)	1,167 (26.1)	1,494 (27.8)	1,285 (23.8)
Demographic factors								
Age (year)	13.2 (2.3)	12.6 (1.9)	13.9 (2.3)	15.5 (1.9)	12.2 (1.6)	13.7 (2.3)	14.2 (2.3)	15.5 (1.9)
Girl, No. (%)	2,935 (46.3)	2,229 (45.7)	2,521 (46.3)	2,626 (50.5)	3,021 (44.9)	2,122 (47.5)	2,572 (47.9)	2,646 (49.1)
Administrative district, No. (%)								
City	1,980 (31.2)	1,693 (34.7)	2,419 (44.5)	2,727 (51.4)	2,013 (29.9)	1,403 (31.4)	2,533 (47.2)	2,870 (53.2)
County	2,257 (35.6)	1,940 (39.8)	1,525 (28.0)	841 (15.9)	2,779 (41.3)	1,656 (37.1)	1,356 (25.2)	772 (14.3)
Town	843 (13.3)	517 (10.6)	656 (12.1)	811 (15.3)	795 (11.8)	552 (12.4)	672 (12.5)	808 (15.0)
Countryside	1,264 (19.9)	729 (14.9)	842 (15.5)	922 (17.4)	1,145 (17.0)	857 (19.2)	811 (15.1)	944 (17.5)
Eye health and behavior								
Myopia, No. (%)	3,738 (58.9)	2,591 (53.1)	3,486 (64.1)	4,053 (76.5)	3,420 (50.8)	2,842 (63.6)	3,537 (65.8)	4,069 (75.4)
Astigmatism, No. (%)	1,889 (29.8)	1,391 (28.5)	1,994 (36.6)	2,630 (49.6)	1,724 (25.6)	1,484 (33.2)	2,101 (39.1)	2,595 (48.1)
Glasses-wearing, No. (%)								
Never	2,752 (43.4)	2,429 (49.8)	2,139 (39.3)	1,336 (25.2)	3,572 (53.1)	1,706 (38.2)	1,981 (36.9)	1,397 (25.9)
Occasionally	1,108 (17.5)	788 (16.2)	871 (16.0)	908 (17.1)	1,029 (15.3)	822 (18.4)	872 (16.2)	952 (17.6)
Always	2,484 (39.2)	1,662 (34.1)	2,432 (44.7)	3,057 (57.7)	2,131 (31.7)	1,940 (43.4)	2,519 (46.9)	3,045 (56.5)
Distance from eyes to the screen, No. (%)								
≤ 33 cm	565 (8.9)	555 (11.4)	621 (11.4)	451 (8.5)	711 (10.6)	394 (8.8)	601 (11.2)	486 (9.0)
34–65 cm	2,580 (40.7)	513 (10.5)	438 (8.0)	262 (4.9)	1,472 (21.9)	1,582 (35.4)	424 (7.9)	315 (5.8)
≥66 cm	3,199 (50.4)	3,811 (78.1)	4,383 (80.5)	4,588 (86.5)	4,549 (67.6)	2,492 (55.8)	4,347 (80.9)	4,593 (85.2)
Eye exercise, No. (%)
0 times/week	1,978 (31.2)	2,341 (48.0)	2,715 (49.9)	3,025 (57.1)	2,915 (43.3)	1,577 (35.3)	2,737 (50.9)	2,830 (52.5)
1–4 times/week	913 (14.4)	892 (18.3)	927 (17.0)	814 (15.4)	1,112 (16.5)	682 (15.3)	894 (16.6)	858 (15.9)
5–6 times/week	932 (14.7)	925 (19.0)	976 (17.9)	752 (14.2)	1,039 (15.4)	676 (15.1)	989 (18.4)	881 (16.3)
≥7 times/week	2,521 (39.7)	721 (14.8)	823 (15.1)	710 (13.4)	1,666 (24.7)	1,532 (34.3)	752 (14.0)	825 (15.3)
Eye drops for foreign body sensation, dry or fatigue eyes, No. (%)								
0 times/day	4,194 (66.1)	4,260 (87.3)	4,552 (83.7)	4,090 (77.2)	5,341 (79.3)	3,169 (71.0)	4,447 (82.8)	4,139 (76.7)
1 times/day	254 (4.0)	239 (4.9)	367 (6.7)	445 (8.4)	271 (4.0)	206 (4.6)	361 (6.7)	467 (8.7)
2 times/day	1,723 (27.2)	247 (5.1)	339 (6.2)	455 (8.6)	978 (14.5)	970 (21.7)	360 (6.7)	456 (8.5)
≥3 times/day	171 (2.7)	131 (2.7)	182 (3.3)	311 (5.9)	142 (2.1)	119 (2.7)	202 (3.8)	332 (6.2)
Lifestyle factors								
Physical activity								
duration (hours/day)	1.2 (0.9)	1.2 (0.9)	1.1 (0.9)	0.9 (0.8)	1.2 (0.9)	1.1 (0.8)	1.1 (0.8)	1.0 (0.9)
Intensity, No. (%)								
Light-intensity	1,636 (25.8)	1,565 (32.1)	2,068 (38.0)	2,462 (46.4)	2,021 (30.0)	1,263 (28.3)	2,073 (38.6)	2,374 (44.0)
Moderate-intensity	4,546 (71.7)	3,218 (66.0)	3,242 (59.6)	2,668 (50.3)	4,574 (67.9)	3,104 (69.5)	3,148 (58.6)	2,848 (52.8)
Vigorous-intensity	162 (2.6)	96 (2.0)	132 (2.4)	171 (3.2)	137 (2.0)	101 (2.3)	151 (2.8)	172 (3.2)
Physically active, No. (%)^c^	3,543 (55.8)	2,120 (43.5)	1,871 (34.4)	1,340 (25.3)	3,289 (48.9)	2,339 (52.4)	1,797 (33.5)	1,449 (26.9)
Sleep time (hours/day)	8.6 (1.1)	8.7 (1.1)	8.3 (1.1)	7.7 (1.0)	8.9 (1.1)	8.5 (1.1)	8.2 (1.1)	7.7 (1.0)
Sleep time, No. (%)								
<8.0 hours/day	677 (10.7)	485 (9.9)	1,190 (21.9)	2,144 (40.4)	379 (5.6)	594 (13.3)	1,333 (24.8)	2,190 (40.6)
8.0–9.9 hours/day	4,449 (70.1)	3,251 (66.6)	3,536 (65.0)	2,871 (54.2)	4,568 (67.9)	3,160 (70.7)	3,421 (63.7)	2,958 (54.8)
≥10.0 hours/day	1,218 (19.2)	1,143 (23.4)	716 (13.2)	286 (5.4)	1,785 (26.5)	714 (16.0)	618 (11.5)	246 (4.6)
Change in diet habit, No. (%)	1,171 (18.5)	797 (16.3)	1,011 (18.6)	1,112 (21.0)	1,182 (17.6)	820 (18.4)	964 (17.9)	1,125 (20.9)
Psychological stress								
Perceived stress score	4.9 (2.9)	5.1 (2.7)	5.6 (2.9)	6.3 (3.1)	4.9 (2.7)	5.1 (2.9)	5.7 (2.9)	6.1 (3.1)
Concerned about COVID-19	1.6 (1.2)	1.6 (1.1)	1.5 (1.1)	1.5 (1.1)	1.6 (1.2)	1.6 (1.2)	1.5 (1.1)	1.5 (1.1)

*COVID, coronavirus disease; NA, not applicable; SD, standard deviation*.

a *Data values were mean (SD), or number (percentage)*.

b *P-values for the overall difference in the related variables across quartiles of the total screen/online-course time from analysis of variance (ANOVA), chi-squared test, or Kruskal-Wallis equality-of-population rank test, as appropriate, were all < 0.001*.

c *Participants were considered physically active if they did moderate or vigorous activity ≥ 4 times/week and ≥ 30 min/day*.

#### Associations of Total Screen/Online-Course Time and Asthenopia Risk

We found that total screen time was positively associated with asthenopia risk (Table 3). Participants in the highest quartile of total screen time have a significantly higher risk of asthenopia (OR = 1.44; 95% CI: 1.23–1.68; *P*_linear−trend_ <0.001) compared to those in the lowest quartile after adjustment for potential confounders. When the students were stratified by gender, the positive association was consistently observed in 11,605 men (Q_4_/Q_1_: 1.45; 1.16–1.8) and 10,361 women (Q_4_/Q_1_: 1.31; 1.06–1.63) (*P*_interaction_ = 0.416). No significant difference was observed between 5,385 graduating students (Q_4_/Q_1_: 1.46; 1.06–2) and 16,581 non-graduating students either (Q_4_/Q_1_: 1.40; 1.17–1.67) (*P*_interaction_ = 0.44). When the total online-course time was assessed separately, the positive association did not materially change (Q_4_/Q_1_: 1.4; 1.18–1.66; *P*_linear−trend_ <0.001). Neither gender nor graduating status significantly modified the positive association.

**Table 3:** The associations [OR (95% CI)] of total screen time/online-course time with the risk of asthenopia^a^.

	**Quartiles of exposure (hours)**				***P* for linear trend^b^**	**Random effects [σ** **(SE)]**^c^		**Residual ICC (95% CI)** ^d^	
	**Q1 (lowest)**	**Q2**	**Q3**	**Q4 (highest)**		**Level 3**	**Level 2**	**Level 3**	**Level 2 | 3**
Total screen time (hours)	≤ 84.0	86.8–175.0	176.4–350.0	≥357.0	NA	NA	NA	NA	NA
No. of participants	6,344	4,879	5,442	5,301	NA	NA	NA	NA	NA
Events, No. (%)	539 (8.50)	363 (7.44)	651 (11.96)	1,094 (20.94)	NA	NA	NA	NA	NA
Model 1^e^	Reference	1.05 (0.90, 1.23)	1.24 (1.07, 1.43)	1.67 (1.44, 1.94)	<0.001	0.143 (0.079)	0.154 (0.042)	0.006 (0.001, 0.051)	0.013 (0.005, 0.034)
**Model 2^f^**	Reference	1.00 (0.86, 1.18)	1.15 (0.99, 1.33)	1.48 (1.27, 1.72)	<0.001	0.412 (0.155)	0.147 (0.043)	0.049 (0.011, 0.184)	0.055 (0.016, 0.176)
**Model 3^g^**	Reference	1.02 (0.87, 1.20)	1.16 (0.999, 1.35)	1.44 (1.23, 1.68)	<0.001	0.382 (0.152)	0.162 (0.043)	0.042 (0.009, 0.173)	0.050 (0.014, 0.161)
**Total online-course time (hours)**	≤ 84.0	85.8–140.0	142.8–280.0	≥ 283.5	NA	NA	NA	NA	NA
**No of participants**	6,732	4,468	5,372	5,394	NA	NA	NA	NA	NA
**Events, No. (%)**	411 (6.11)	487 (10.90)	737 (13.72)	1,012 (18.76)	NA	NA	NA	NA	NA
**Model 1^e^**	Reference	1.13 (0.97, 1.32)	1.30 (1.11, 1.52)	1.45 (1.23, 1.71)	<0.001	0.118 (0.079)	0.160 (0.043)	0.004 (0.000, 0.054)	0.012 (0.005, 0.029)
**Model 2^f^**	Reference	1.13 (0.97, 1.31)	1.27 (1.09, 1.49)	1.42 (1.20, 1.68)	<0.001	0.407 (0.154)	0.147 (0.043)	0.048 (0.011, 0.181)	0.054 (0.015, 0.173)
**Model 3^g^**	Reference	1.15 (0.99, 1.34)	1.29 (1.10, 1.51)	1.40 (1.18, 1.66)	<0.001	0.376 (0.149)	0.159 (0.044)	0.041 (0.009, 0.168)	0.048 (0.014, 0.157)

*CI, confidence interval; ICC, interclass correlation; NA, not applicable; OR, odds ratio; Q, quartile; SE, standard error*.

a *All the models were constructed by using mixed-effects logistic regression with individuals (level 1) nested in school (level 2) and province (level 3)*.

b *P for linear trend was calculated by using the exposure of interest as a continuous variable*.

c, d *From the models with exposure of interest in quartiles*.

e *Model 1: adjusted age, gender, administrative district, physical activity, sleep time, myopia, astigmatism, and glasses-wearing status*.

f *Model 2: adjusted variables in model 1, lying down or lying on the stomach while watching a screen, and distance from eyes to screen*.

g *Model 3: adjusted variables in model 2, rest time between classes, eye exercise, and eye drops for foreign body sensation, dry or fatigued eyes*.

A 100-h increment in total screen/online-course time was associated with an increment of 9% (1.09; 1.07–1.12) and 11% (1.11; 1.06–1.17) risk of asthenopia (Table 4). Mediation analysis showed that the proportions mediated by psychological stress were 23.5 and 38.1%, respectively.

**Table 4:** Multi-level multivariable-adjusted associations [OR (95% CI)] of the total screen/online-course time and related covariates with asthenopia risk^a^.

**Variable**	**Comparison**	**Total screen time**	**Total online-course time**
Total screen or online-course time	↑ 100-h	1.09 (1.07, 1.12)	1.11 (1.06, 1.17)
Age	↑ 1-year	1.15 (1.11, 1.18)	1.15 (1.12, 1.19)
Gender	Boys	Reference	Reference
	Girls	1.19 (1.09, 1.29)	1.19 (1.09, 1.30)
District	City	Reference	Reference
	County	1.04 (0.89, 1.20)	1.04 (0.90, 1.21)
	Town	1.12 (0.96, 1.31)	1.12 (0.96, 1.32)
	Countryside	1.06 (0.90, 1.23)	1.06 (0.91, 1.23)
Physically active	No	Reference	Reference
	Yes	0.92 (0.83, 1.02)	0.92 (0.83, 1.01)
Sleep time	<8 h/day	1.42 (1.28, 1.58)	1.43 (1.29, 1.59)
	8.0–9.9 h/day	Reference	Reference
	≥ 10 h/day	0.96 (0.81, 1.13)	0.92 (0.81, 1.13)
Myopia	No	Reference	Reference
	Yes	1.51 (1.27, 1.81)	1.51 (1.26, 1.80)
Astigmatism	No	Reference	Reference
	Yes	1.59 (1.45, 1.75)	1.60 (1.45, 1.76)
Glass-wearing	Never	Reference	Reference
	Occasionally	1.15 (0.95, 1.38)	1.15 (0.95, 1.38)
	Always	0.98 (0.82, 1.17)	0.98 (0.82, 1.17)
Lying down or lying on the stomach while watching a screen	Never	Reference	Reference
	Occasionally	1.51 (1.36, 1.69)	1.53 (1.37, 1.71)
	Often	2.47 (2.11, 2.88)	2.59 (2.22, 3.02)
	Always	4.72 (3.44, 6.50)	4.98 (3.63, 6.84)
Distance from eyes to the screen	≤ 33 cm	Reference	Reference
	34–65 cm	0.55 (0.42, 0.72)	0.54 (0.41, 0.71)
	≥ 66 cm	1.25 (1.06, 1.46)	1.26 (1.08, 1.48)
Rest time between classes	↑ 20 min	0.96 (0.91, 0.999)	0.95 (0.91, 0.99)
Eye exercise	0 times/week	Reference	Reference
	1 to 4 times/week	0.81 (0.71, 0.92)	0.80 (0.70, 0.91)
	5 to 6 times/week	0.59 (0.50, 0.68)	0.57 (0.49, 0.67)
	≥ 7 times/week	0.69 (0.59, 0.80)	0.67 (0.58, 0.78)
Eye drops for foreign body sensation to dry or fatigued eyes	0 times/day	Reference	Reference
	1 times/day	1.46 (1.24, 1.73)	1.46 (1.24, 1.73)
	2 times/day	1.59 (1.37, 1.83)	1.57 (1.36, 1.82)
	> 2 times/day	2.50 (2.09, 2.99)	2.48 (2.07, 2.97)
Random effects [σ (SE)]
Level 3	NA	0.397 (0.155)	0.368 (0.150)
Level 2	NA	0.161 (0.043)	0.169 (0.043)
Residual ICC (95% CI)
Level 3	NA	0.045 (0.010, 0.180)	0.039 (0.008, 0.168)
Level 2 | 3	NA	0.053 (0.015, 0.170)	0.047 (0.013, 0.154)

*CI, confidence interval; ICC, interclass correlation; NA, not applicable; OR, odds ratio; SE, standard error*.

a *All the models were constructed by using mixed-effects logistic regression with individuals (level 1) nested in school (level 2) and province (level 3). The exposure of interest was modeled as a continuous variable*.

### Associations of Other Covariates With Asthenopia Risk

The associations between all the adjusted covariates from model 3 in Table 3 and asthenopia risk while the exposure was modeled as a continuous variable (per increment of 100-h) were presented in Table 4.

Age (1.15; 1.11–1.18 for every 1-year increment), women (1.19; 1.09–1.29), having myopia (1.51; 1.27–1.81) or astigmatism (1.59; 1.45–1.75) or bad habits, e.g., lying down or lying on their stomachs when watching screens (1.61; 1.5–1.73 for every 1-level increment), keeping eyes a greater distance (≥ 66 vs. ≤ 33 cm) from the screen (1.25; 1.06–1.46), and using eye drops (1.33; 1.27–1.4 for every 1-level increment) were all positively associated with asthenopia risk. While keeping distance between eyes and screen as 34–65 cm (vs. ≤ 33 cm) (0.55;0.42–0.72), increased rest time between classes (0.96;0.91–0.999 for every 20-min increment) and increased frequency of eye exercise (0.85;0.81–0.89 for every 1-level increment) was inversely associated with asthenopia risk. When examining the total online-course time separately, all the conclusions remained.

**Figure 1:**
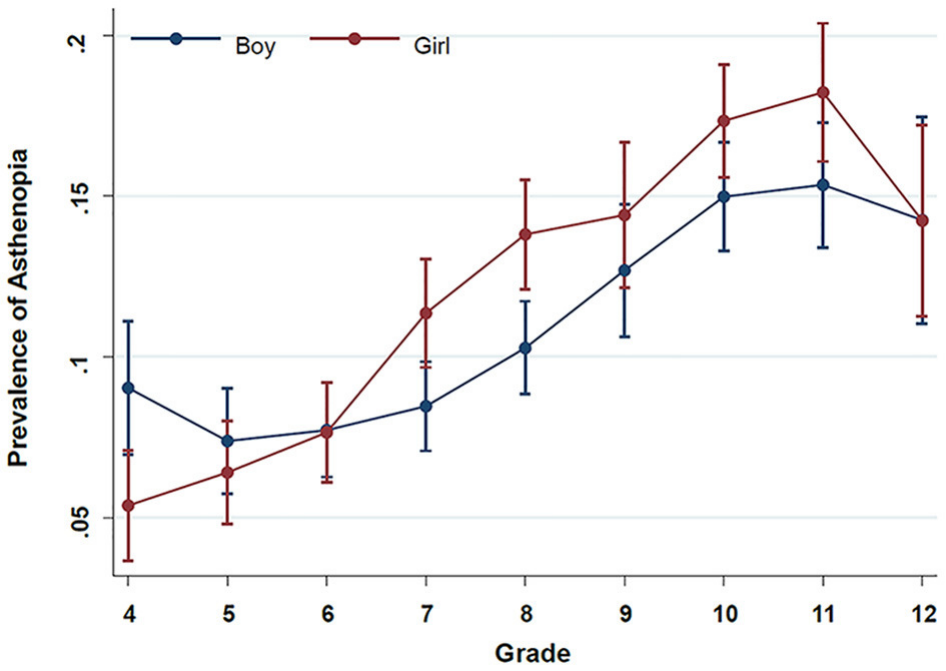
Prevalence of asthenopia with 95% confidence interval across grade stratified by gender. The prevalence of asthenopia was estimated from a marginal logistic regression model using the total screen time as the exposure and with the adjustment for the grade, gender, grade*gender, administrative district (city, county, town, or countryside), physical activity (active vs. not active), and sleep time (<8, 8–9.9, ≥10 h), myopia (yes vs. no), astigmatism (yes vs. no), and glasses-wearing status (never, occasionally, or always), lying down or lying on the stomach while watching a screen (never, occasionally, often, or always), distance from eyes to screen ( ≤ 33, 34–65, or ≥66 cm), rest time between classes, eye exercise (0, 1–4, 5–6, or ≥7 times/week), and eye drops for foreign body sensation, dry or fatigue eyes (0, 1, 2, or ≥3 times/day).

Since grade was positively correlated with age (Pearson *r* = 0.95), when we explored the association between grade and asthenopia risk in the model using total screen time as the exposure, we excluded age from the models. Each 1-grade increment was associated with an increased risk of 14% of asthenopia (1.14; 1.01–1.18), and a significant interaction between grade and gender was identified (*P*_interaction_ = 0.029). The predicted prevalence of asthenopia across grades stratified by gender is shown in Figure 1. The phenomenon that women had a higher risk of asthenopia than men was observed only among sixth graders and above, while among fifth graders and below, women had a lower risk than men. Again, all the conclusions were not materially changed in the model with the total online-course time as the exposure.

## Discussion

### Main Findings

Our study is the first report demonstrating prolonged total screen time and online-course time were associated with increased risk of asthenopia among school-aged children in eastern China during the COVID-19 self-quarantine period. These associations were partially mediated by psychological stress. The positive associations were consistently observed among men and women, as well as in graduating and non-graduating students. Other “risk” factors included age, being a woman, having myopia or astigmatism, bad habits when watching screens, keeping eyes out of a reasonable distance from the screen, and using eye drops. We presumed eye drop use as a surrogate of eye dryness or strain. Moreover, increased rest time between classes and increased frequency of eye exercise is beneficial.

In this study, school-aged children in eastern China during the COVID-19 self-quarantine period with asthenopia reported various symptoms (e.g., eye strain and eye pain) related to prolonged use of eyes. Those eye discomforts could result in blurred vision, lower learning speed, and introduce errors, which profoundly impact their physical and mental development (26). Therefore, finding risky/protective factors of asthenopia and establishing prevention strategies were of significant clinical impact and public health value.

A 10-year follow-up study documented that prolonged screen time, use of lenses and psychosocial factors were significantly related to increased asthenopia (27). The positive association between increased screen hours and asthenopia risk was also documented in some studies (16, 28, 29), but not in another study conducted among bank workers in Italy (30).

Our evidence suggested that asthenopia increased with age among school-aged children. Most likely, the extended time with near work in the older age group requires more vergence and accommodative effort, both related to asthenopia symptoms (31, 32). Besides, in our study, children with myopia and astigmatism were more likely to have asthenopia than their counterparts. Interestingly, women were more likely to have asthenopia than men among six graders and above. In contrast, men were more likely to have asthenopia than women among fifth graders and below, which might be due to differences in physical activity and genetic susceptibility, and needs further confirmation.

During the COVID-19 pandemic, the public tended to experience more anxiety and loneliness (17, 33). Being self-isolated at home and lacking face-to-face communication with teachers and classmates for such an extended period have been suggested to generate psychological disorders such as depression and anxiety (34, 35).

Previous studies found that psychological stress was positively related to asthenopia, particularly among digital screen-using populations, including high-tech workers (36) and college students (28, 37). In our study, psychological stress was evaluated by perceived stress scores and concerns about coronavirus infection. Higher perceived stress was observed in higher levels of the screen time or online-course time group. The average perceived stress score of participants with asthenopia was significantly higher compared to participants without asthenopia.

Frequently blinking is vital for producing and maintaining tear film. Staring at a digital screen for hours could reduce blink rate and tear film instability, leading to eye pain or dryness (38, 39). Additionally, perceived stress is associated with somatization, which could aggravate eye discomfort (40, 41). However, higher quartiles of the screen time and online-course time groups, who might have more appropriate access to COVID-19 information from media, showed fewer concerns about coronavirus infection.

According to our findings, asthenopia prevalence was 12.1% in this group of Chinese children with a mean age of 13.8 years, which was much lower than the results from Xi'an college students (57.0%) (28) and Shanghai college students (53.3%) (16). Moreover, our prevalence was lower than that in previously published studies (Supplementary Table 3) in children as well, e.g., studies from India (8, 9), Brazil (10), Italy (11), England (12), Australia (13), and Sweden (14, 15) ranging from 12.4–57.9%. The pooled prevalence of asthenopia in children under 20 years old determined by a meta-analysis was 19.7% (42).

Although further research is required, the low prevalence in Chinese children reported in our study might be related to the fact that we used a stricter definition based on a validated tool. In previous studies, definitions of asthenopia primarily included: (1) self-reported eye strain; (2) the presence of one or more asthenopia symptoms; or (3) a high frequency of feeling less stringent asthenopia symptoms. Also, our large sample size made our estimation of asthenopia prevalence more reliable.

### Study Strengths and Limitations

This study has several strengths. First, our study was one of the largest cross-sectional studies among children, to examine asthenopia and its related risky/protective factors during the COVID-19 pandemic period in China. Second, our study questionnaire lasted for more than one month, which provided a relatively stable snapshot of the asthenopia status of children. Third, eastern China, compared to other regions, has the highest computer coverage rate, which can provide great power with the most prevalent cases. Fourth, the asthenopia data were collected using a validated CVS-Q questionnaire, and its translated Chinese version was re-validated in our pilot study.

Our study also has some limitations. First, like all other observational studies, its strength for justifying a causal relationship is limited. However, it can serve as a foundation for future prospective studies among extended administrative areas with increased sample sizes. Second, we could not rule out residual confounding due to unmeasured variables. Third, two exposures were self-reported and subjective, and measurement error is inevitable. However, it is most likely to be random and may attenuate any possible associations. The measurement could be improved by advanced techniques (e.g., Actigraph) in future studies. Fourth, this study did not include an analysis of genetic susceptibilities relating to detailed mechanisms of asthenopia development.

### Implications for Research and Clinical Interventions

#### Considering the Results Above, We Suggest Some Strategies for Asthenopia Prevention

1. Keeping good eye care habits such as reducing screen time and online-course time, keeping the eyes at a proper distance from the screen (34–65 cm), participating in some self-help program of eye exercise, and taking regular clinical eye care.
2. Maintaining a healthy lifestyle includes (but is not limited to) being physically active, having a balanced diet, and having enough sleep.
3. Being emotionally stable and maintaining positive mental health.

## Conclusion

Being self-quarantined and taking online courses have profoundly changed the learning modes of millions of students worldwide, but its adverse effects on eye health have been largely overlooked. This study suggests that prolonged screen time, online-course time, and psychological stress can significantly increase asthenopia risk. These factors should be considered when it comes to online curriculum schedules and educational policy development.

## Data Availability Statement

The raw data supporting the conclusions of this article will be made available by the authors, without undue reservation.

## Ethics Statement

The studies involving human participants were reviewed and approved by the Ethics Committee of Shanghai Ninth Hospital, School of Medicine, Shanghai Jiao Tong University. Written informed consent to participate in this study was provided by the participants' legal guardian/next of kin.

## Author Contributions

LL, JZha, MC, and JZho conceived the study and its design. LL, JZho, JZha, MC, RJ, and ZL took responsibility for the literature research. JZho, JZha, LL, MC, RJ, ZL, XL, QC, LZ, JS, YW, WZ, JC, and PX contributed to data acquisition. LL, PX, and JZho contributed to data analysis and interpretation. LL and JZho vetted all the results and provided administrative support for the project, had full access to the data, and took responsibility for the integrity of the data and accuracy of the analysis. LL and PX wrote the first draft of the manuscript. LL, JZho, JZha, MC, RJ, ZL, XL, QC, JS, YW, WZ, and JC prepared all the tables and figures. LL, PX, and MC managed the supplements. All authors read and approved the final manuscript.

## Funding

This study was supported by the National Natural Science Foundation of China (Grant No. 81670892) and the Innovative Research Team of High-level Local Universities in Shanghai (Grant No. SSMU-ZDCX20180401).

## Conflict of Interest

The authors declare that the research was conducted in the absence of any commercial or financial relationships that could be construed as a potential conflict of interest.

## Publisher's Note

All claims expressed in this article are solely those of the authors and do not necessarily represent those of their affiliated organizations, or those of the publisher, the editors and the reviewers. Any product that may be evaluated in this article, or claim that may be made by its manufacturer, is not guaranteed or endorsed by the publisher.

## Abbreviations

CVS-Q: Chinese version of computer vision syndrome questionnaire
CI: confidence interval
SARS-CoV-2: severe acute respiratory syndrome coronavirus 2
COVID-19: 2019 novel coronavirus disease
IRT: item response theory
SD: standard deviation
OR: odds ratio.
