# Supplementary material for: Contribution of Total Screen/Online-Course Time to Asthenopia in Children During COVID-19 Pandemic via Influencing Psychological Stress
Source: Front Public Health. 2021 Dec 1;9:736617. doi: 10.3389/fpubh.2021.736617 (PMC8671164; doi:10.3389/fpubh.2021.736617)
Supplement: Supplementary file 1 [file Table_1.DOCX]

**Supplementary Table 1.** The associations [OR (95% CI)] of quartiles of total screen time or online-course time with risk of asthenopia ^a^

|  | **Quartiles of exposure (hours)** | | | | ***p* for linear trend** ^b^ |
| --- | --- | --- | --- | --- | --- |
|  | **Q1 (lowest)** | **Q2** | **Q3** | **Q4 (highest)** |  |
| Total screen time (hours) | ≤ 84.0 | 86.8-175.0 | 176.4-350.0 | ≥ 357.0 | NA |
| No of participants | 6344 | 4879 | 5442 | 5301 | NA |
| Events, No. (%) | 539 (8.50) | 363 (7.44) | 651 (11.96) | 1094 (20.64) | NA |
| Model 1 ^c^ | Reference | 1.07 (0.92, 1.25) | 1.31 (1.13, 1.51) | 1.77 (1.53, 2.05) | <0.001 |
| Model 2 ^d^ | Reference | 1.02 (0.87, 1.19) | 1.21 (1.05, 1.39) | 1.56 (1.35, 1.81) | <0.001 |
| Model 3 ^e^ | Reference] | 1.04 (0.88, 1.22) | 1.22 (1.06, 1.42) | 1.53 (1.32, 1.78) | <0.001 |
| Total online-course time (hours) | ≤ 84.0 | 85.8-140.0 | 142.8-280.0 | ≥ 283.5 | NA |
| No of participants | 6732 | 4468 | 5372 | 5394 | NA |
| Events, No. (%) | 411 (6.11) | 487 (10.90) | 737 (13.72) | 1012 (18.76) | NA |
| Model 1 ^c^ | Reference | 1.16 (1.00, 1.35) | 1.40 (1.21, 1.62) | 1.56 (1.33, 1.82) | <0.001 |
| Model 2 ^d^ | Reference | 1.16 (1.00, 1.34) | 1.37 (1.18, 1.59) | 1.53 (1.31, 1.79) | <0.001 |
| Model 3 ^e^ | Reference | 1.19 (1.02, 1.38) | 1.39 (1.20, 1.61) | 1.52 (1.29, 1.78) | <0.001 |

CI = confidence interval; NA = not applicable; OR = odds ratio; Q = quartile.

^a^ All the models were constructed by using ordinary logistic regression with province adjusted.

^b^ *p* for linear trend was calculated by using the exposure of interest as a continuous variable.

^c^ Model 1: adjusted age, sex, administrative district (city, county, town, or countryside), physical activity (active vs. not active), sleep time (< 8, 8.0-9.9, ≥ 10 hours), myopia (yes vs. no), astigmatism (yes vs. no), and glasses-wearing status (never, occasionally, or always).

^d^ Model 2: adjusted variables in model 1, lying down or lying on the stomach while watching a screen (never, occasionally, often, or always), and distance from eyes to screen (≤ 33, 34-65, or ≥ 66 cm).

^e^ Model 3: adjusted variables in model 2, rest time between classes, eye exercise (0, 1 to 4, 5 to 6, or ≥ 7 times/week), and eye drops for foreign body sensation, dry or fatigue eyes (0, 1, 2, or ≥3 times/day).
