# Supplementary material for: Contribution of Total Screen/Online-Course Time to Asthenopia in Children During COVID-19 Pandemic via Influencing Psychological Stress
Source: Front Public Health. 2021 Dec 1;9:736617. doi: 10.3389/fpubh.2021.736617 (PMC8671164; doi:10.3389/fpubh.2021.736617)
Supplement: Supplementary file 2 [file Table_2.DOCX]

**Supplementary Table 2.** The associations [OR (95% CI)] of continuous total screen time or online-course time as well as related covariates with risk of asthenopia ^a^

| **Variable** | **Comparison** | **Total screen time** | **Total online to course time** |
| --- | --- | --- | --- |
| Total screen/online, course time | ↑ 100-hour | 1.10 (1.07, 1.13) | 1.08 (1.05, 1.12) |
| Age | ↑ 1 year old | 1.17 (1.14, 1.20) | 1.17 (1.15, 1.20) |
| Sex | Boys | Reference | Reference |
|  | Girls | 1.18 (1.08, 1.29) | 1.18 (1.08, 1.29) |
| District | City | Reference | Reference |
|  | County | 1.03 (0.89, 1.19) | 1.03 (0.89, 1.20) |
|  | Town | 1.15 (0.98, 1.33) | 1.15 (0.99, 1.34) |
|  | Countryside | 1.07 (0.92, 1.24) | 1.07 (0.93, 1.24) |
| Physically active | No | Reference | Reference |
|  | Yes | 0.92 (0.83, 1.01) | 0.91 (0.82, 1.00) |
| Sleep time | < 8 hours/day | 1.43 (1.29, 1.58) | 1.44 (1.30, 1.60) |
|  | 8.0-9.9 hours/day | Reference | Reference |
|  | ≥ 10 hours/day | 0.92 (0.78, 1.08) | 0.92 (0.79, 1.09) |
| Myopia | No | Reference | Reference |
|  | Yes | 1.51 (1.26, 1.80) | 1.50 (1.26, 1.79) |
| Astigmatism | No | Reference | Reference |
|  | Yes | 1.59 (1.45, 1.75) | 1.60 (1.46, 1.77) |
| Glasses, wearing | Never | Reference | Reference |
|  | Occasionally | 1.16 (0.97, 1.40) | 1.16 (0.97, 1.40) |
|  | Always | 0.99 (0.82, 1.18) | 0.99 (0.83, 1.19) |
| Lying down or lying on the stomach while watching a screen | Never | Reference | Reference |
|  | Occasionally | 1.51 (1.35, 1.68) | 1.53 (1.37, 1.70) |
|  | Often | 2.45 (2.10, 2.86) | 2.58 (2.21, 3.00) |
|  | Always | 4.70 (3.42, 6.46) | 4.97 (3.62, 6.82) |
| Distance from eyes to the screen | ≤ 33 cm | Reference | Reference |
|  | 34-65 cm | 0.52 (0.40, 0.68) | 0.51 (0.38, 0.66) |
|  | ≥ 66 cm | 1.27 (1.08, 1.49) | 1.29 (1.10, 1.52) |
| Rest time between classes | ↑ 20 minutes | 0.95 (0.91, 0.997) | 0.95 (0.91, 0.99) |
| Eye exercise | 0 times/week | Reference | Reference |
|  | 1 to 4 times/week | 0.81 (0.71, 0.92) | 0.80 (0.70, 0.90) |
|  | 5 to 6 times/week | 0.59 (0.51, 0.69) | 0.58 (0.50, 0.67) |
|  | ≥ 7 times/week | 0.69 (0.59, 0.80) | 0.67 (0.58, 0.78) |
| Eye drops for foreign body sensation, dry or fatigued eyes | 0 times/day | Reference | Reference |
|  | 1 times/day | 1.46 (1.23, 1.72) | 1.45 (1.23, 1.72) |
|  | 2 times/day | 1.57 (1.36, 1.81) | 1.55 (1.34, 1.79) |
|  | > 2 times/day | 2.48 (2.08, 2.97) | 2.46 (2.06, 2.95) |

CI = confidence interval; OR= odds ratio.

^a^ The results were from model 3 in Supplementary Table 1, which were also constructed by using ordinary logistic regression with province adjusted. The exposure of interest was modeled as a continuous variable.
