# Supplementary material for: Contribution of Total Screen/Online-Course Time to Asthenopia in Children During COVID-19 Pandemic via Influencing Psychological Stress
Source: Front Public Health. 2021 Dec 1;9:736617. doi: 10.3389/fpubh.2021.736617 (PMC8671164; doi:10.3389/fpubh.2021.736617)
Supplement: Supplementary file 3 [file Table_3.DOCX]

**Supplementary Table 3.** Prevalence of asthenopia of children under 20 years old in previously published studies

| **Sources** | **Country** | **Design** | **Group** | **Sample size** | **Age ^a^** | **Boys (n, %)** | **Prevalence (n, %)** |
| --- | --- | --- | --- | --- | --- | --- | --- |
| **Ip *et al*. (2006)** (1) | Australia | Cross-sectional | ─ | 1462 | 6 (NA) | NA | 219 (15.0) |
| **Sterner *et al.* (2006)** (2) | Sweden | Prospective cohort | First examination | 72 | 5.8-10.0 | 43 (59.7) | 19 (26.4) |
|  |  |  | Second examination | 59 | 7.8-11.8 | 34 (57.6) | 14 (23.7) |
| **Abdi *et al.* (2008)** (3) | Sweden | Cross-sectional | ─ | 216 | 6-16 | 105 (48.6) | 50 (23.1) |
| **Tiwari *et al.* (2011)** (4) | India | Cross-sectional comparative | Control group | 569 | 12.4 (7.0) | NA | 137 (24.1) |
|  |  |  | Gem polishing group | 432 | 11.2 (2.0) | 205 (47.4) | 139 (32.2) |
| **Tiwari (2013)** (5) | India | Cross-sectional comparative | Control group | 160 | 11.0 (1.5) | NA | 20 (12.4) |
|  |  |  | Labor group | 139 | 10.8 (1.5) | 56 (40.3) | 36 (25.9) |
| **Vilela *et al.* (2015)** (6) | Brazil | Cross-sectional | ─ | 964 | 6-16 | 532 (55.2) | 238 (24.7) |
| **Rechichi *et al.* (2017)** (7) | Italy | Cross-sectional comparative | Control group | 85 | 3-10 | 36 (42.4) | 23 (27.1) |
|  |  |  | Video game group | 235 | 3-10 | 123 (52.3) | 136 (57.9) |
| **Ichhpujani *et al.* (2019)(8)** | England | Cross-sectional | ─ | 576 | 11-17 | 349 (60.6) | 103 (17.9) |

Abbreviations: NA, not available; SD, standard deviation

^a^ Data were means (SDs) or “minimums to maximums”

**REFERENCES**

1. Ip JM, Robaei D, Rochtchina E, Mitchell P. Prevalence of eye disorders in young children with eyestrain complaints. Am J Ophthalmol. (2006). 142(3):495-7.

2. Sterner B, Gellerstedt M, Sjostrom A. Accommodation and the relationship to subjective symptoms with near work for young school children. Ophthalmic Physiol Opt. (2006). 26(2):148-55.

3. Abdi S, Lennerstrand G, Pansell T, Rydberg A. Orthoptic findings and asthenopia in a population of Swedish schoolchildren aged 6 to 16 years. Strabismus. (2008). 16(2):47-55.

4. Tiwari RR, Saha A, Parikh JR. Asthenopia (eyestrain) in working children of gem-polishing industries. Toxicol Ind Health. (2011). 27(3):243-7.

5. Tiwari RR. Eyestrain in working children of footwear making units of Agra, India. Indian Pediatr. (2013). 50(4):411-3.

6. Vilela MA, Castagno VD, Meucci RD, Fassa AG. Asthenopia in schoolchildren. Clin Ophthalmol. (2015). 9:1595-603.

7. Rechichi C, De Moja G, Aragona P. Video game vision syndrome: a new clinical picture in children? J Pediatr Ophthalmol Strabismus. (2017). 54(6):346-55.

8. Ichhpujani P, Singh RB, Foulsham W, Thakur S, Lamba AS. Visual implications of digital device usage in school children: a cross-sectional study. BMC Ophthalmol. (2019). 19(1):76.
