# Supplementary figures and images for: Contribution of Total Screen/Online-Course Time to Asthenopia in Children During COVID-19 Pandemic via Influencing Psychological Stress
Source: Front Public Health. 2021 Dec 1;9:736617. doi: 10.3389/fpubh.2021.736617 (PMC8671164; doi:10.3389/fpubh.2021.736617)

**Supplementary Figure 1** Flow chart of this study


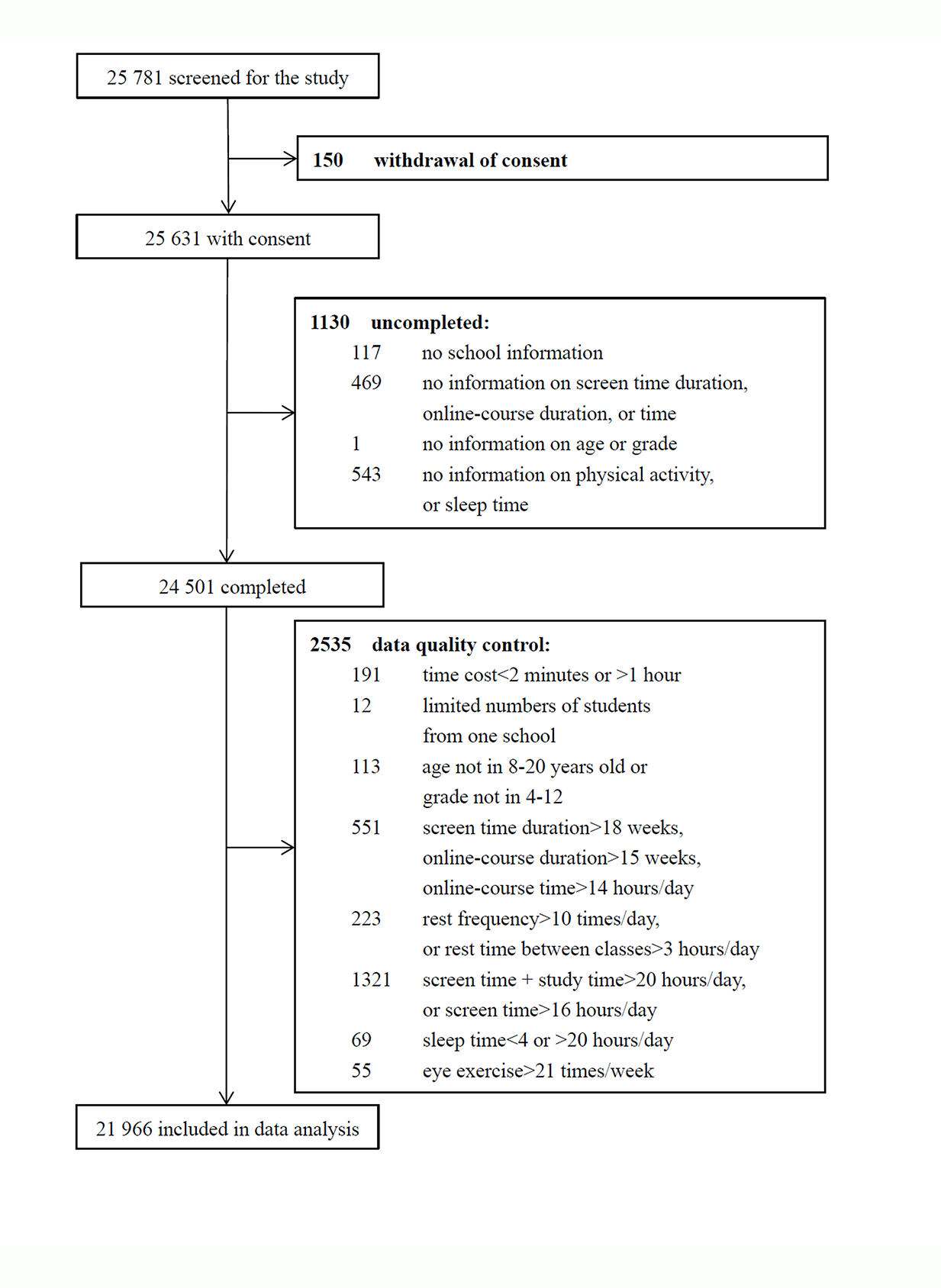

Supplement: Supplementary file 4 [file Data_Sheet_1.DOCX]
