# Supplementary material for: Contribution of Total Screen/Online-Course Time to Asthenopia in Children During COVID-19 Pandemic via Influencing Psychological Stress
Source: Front Public Health. 2021 Dec 1;9:736617. doi: 10.3389/fpubh.2021.736617 (PMC8671164; doi:10.3389/fpubh.2021.736617)
Supplement: Supplementary file 5 [file Data_Sheet_2.DOCX]

**Supplementary Method 1.** Online-course Student Eye Use and Eye Strain Questionnaire

**Online-course Student Eye Use and Eye Strain Questionnaire**

**Informed Consent**

Dear students,

Greetings!

It has been a difficult time due to the COVID-19 pandemic, during which your only learning mode option has been taking online courses at home. Such a learning mode may pose varying degrees of impact on your sight and eye health. We would like you to spare ten minutes to complete this questionnaire. The data will be analyzed only for classification and assessment. A follow-up and monitoring of your eye health will continue with further investigations. The questionnaire is solely for the reference of professionals and health education authorities. Strict confidentiality of the data is guaranteed. No individual information will be disclosed in the published report. There is no right or wrong answer to the questions. Authenticity is all that matters. Please rest assured and provide your honest answers. We appreciate your cooperation. (All blanks are to be filled with single answer choices or arabic numerals except the one for school name, which requires a specific input.)

Do you consent to fill out this questionnaire?

□Yes

□No

I. General Information

1. School name

2. Grade

□ 4th grade, primary school

□ 5th grade, primary school

□ 6th grade, primary school

□ 1st grade, junior high school

□ 2nd grade, junior high school

□ 3rd grade, junior high school

□ 1st grade, high school

□ 2nd grade, high school

□ 3rd grade, high school

3. Student ID

4. Gender

□ Male

□ Female

5. Age (in number of years)

6. Current residence (administrative district)

□ Urban

□ County

□ Town

□ Countryside

7. Are you living with

□ Myopia

□ Hyperopia

□ None

8. Are you living with astigmatism?

□ Yes

□ No

9. Do you wear eyeglasses (including glasses with frames, contact lenses, and orthokeratology lenses)?

□ Always

□ Occassionally

□ Never

II. Eye Use and Health Awareness Questionnaire

Prompt: Please provide information about your behavior patterns during the pandemic/self-quarantine period

1. How many weeks have you been self-quarantined?

2. How many weeks have you been taking online courses?

3. How many hours do you spend on online courses each day?

4. Which equipment do you use for online courses?

□ Projector

□ Television

□ Personal computer

□ iPad

□ Mobile phone

5. How many rests do you take between online courses per day?

6. How many hours do you usually rest between classes?

7. What’s your rest activity between online courses?

□ Looking out of a window

□ Using cellphone

□ Reading books

□ Closing eyes

□ Others

8. How long is your screen time (including time for online courses, homework, games, movies, and TV shows, etc.)? (hours/day)

9. How long is your study time without a screen (including time for homework, reading, and writing)? (hours/day)

10. How long is your study time without a screen (including time for homework, reading, and writing)? (days/week)

11. How many hours do you exercise per day?

12. How many times do you exercise per week?

13. What is the intensity of your exercise?

□ Light intensity

□ Moderate intensity

□ Vigorous intensity

14. Where do you exercise?

□ Outdoors

□ Indoors

□ Both

15.How many hours do you sleep per day?

16. During the pandemic, has there been any change in your diet habit?

□ Yes

□ No

17. Has there been any change to the amount of fruit and vegetables you take?

□ Increase

□ Decrease

□ No change

18. Has there been any change to the amount of meat, eggs, poultry and dairy products you take?

□ Increase

□ Decrease

□ No change

19. Has there been any change to your staple food?

□ Increase

□ Decrease

□ No change

20. Do you lie down or on your stomach while watching a screen?

□ Never

□ Occassionally

□ Often

□ Always

21. What is the light intensity while you are using a screen?

□ Strong

□ Moderate

□ Dim

22. What is the distance from your eyes to the screen while taking online courses?

□ ≤33 cm

□ 34-65 cm

□ ≥66 cm

23. What do you think is the impact of online-course on your eye health?

□ I don’t know.

□ I don’t think there is any impact.

□ I know it may cause dry eyes, myopia, or eye strain.

24. Has anyone told you recently about ways to protect your eyes while taking online courses?

□ Yes

□ No

25. If yes, where do the information and knowledge come from [multiple-choice]

□ Teachers and school

□ Parents

□ Classmates and friends

□ Self-learning from books and the internet

□ Other sources

26. Have you been doing eye exercise recently?

□ Yes

□ No

27. How often do you do eye exercise? (times/week)

28. Have you been using eye drops for foreign body sensation, dry or fatigue eyes recently?

□ Yes

□ No

29. If you have been using eye drops for foreign body sensation, dry or fatigue eyes, how often do you use them? (times/day)

30. Think back to the past month, how often did you experience the following situations?

| 1. Feeling that you were unable to control the important things in your life. | | | | |
| --- | --- | --- | --- | --- |
| Never □ | Seldom □ | Occasionally □ | Often □ | Always □ |
| 2. Feeling confident about your ability to handle your personal problems. | | | | |
| Never □ | Seldom □ | Occasionally □ | Often □ | Always □ |
| 3. Feeling that things were going your way. | | | | |
| Never □ | Seldom □ | Occasionally □ | Often □ | Always □ |
| 4. Feeling difficulties were piling up so high that you could not overcome them. | | | | |
| Never □ | Seldom □ | Occasionally □ | Often □ | Always □ |

31. In your opinion, the above pressure mainly came from:

| 1. Parents keep an eye on your study | | | | |
| --- | --- | --- | --- | --- |
| None □ | A little □ | Some □ | A lot □ | Extremely high □ |
| 2. Online-courses bring poorer academic performance than traditional classes | | | | |
| None □ | A little □ | Some □ | A lot □ | Extremely high □ |
| 3. Unable to communicate with classmates face to face | | | | |
| None □ | A little □ | Some □ | A lot □ | Extremely high □ |
| 4. Unable to communicate with teachers face to face | | | | |
| None □ | A little □ | Some □ | A lot □ | Extremely high □ |

32. For the past one month, how were you concerned about yourself or your family members getting infected by COVID-19?

□ Not at all concerned.

□ Little concerned.

□ Somehow concerned.

□ Very concerned.

□ Extremely concerned.

III. Eye Strain Questionnaire

1. Indicate whether you experienced any of the following symptoms during the past month. If yes, mark frequency for each symptom. If no, mark “Never.”

| Frequency  Symptom | Always | Often | Occasionally | Never |
| --- | --- | --- | --- | --- |
| Burning | □ | □ | □ | □ |
| Itching | □ | □ | □ | □ |
| Feeling of a foreign body | □ | □ | □ | □ |
| Tearing | □ | □ | □ | □ |
| Excessive blinking | □ | □ | □ | □ |
| Eye redness | □ | □ | □ | □ |
| Eye pain | □ | □ | □ | □ |
| Heavy eyelids | □ | □ | □ | □ |
| Dryness | □ | □ | □ | □ |
| Blurred vision | □ | □ | □ | □ |
| Double vision | □ | □ | □ | □ |
| Difficulty focusing for near vision | □ | □ | □ | □ |
| Increased sensitivity to light | □ | □ | □ | □ |
| Colored halos around objects | □ | □ | □ | □ |
| Feeling that sight is worsening | □ | □ | □ | □ |
| Headache | □ | □ | □ | □ |

2. Indicate whether you experienced any of the following symptoms during the past month. If yes, mark severity for each symptom. If no, mark “Never.”

| Frequency  Symptom | Never | Severe | Moderate | Mild |
| --- | --- | --- | --- | --- |
| Burning | □ | □ | □ | □ |
| Itching | □ | □ | □ | □ |
| Feeling of a foreign body | □ | □ | □ | □ |
| Tearing | □ | □ | □ | □ |
| Excessive blinking | □ | □ | □ | □ |
| Eye redness | □ | □ | □ | □ |
| Eye pain | □ | □ | □ | □ |
| Heavy eyelids | □ | □ | □ | □ |
| Dryness | □ | □ | □ | □ |
| Blurred vision | □ | □ | □ | □ |
| Double vision | □ | □ | □ | □ |
| Difficulty focusing for near vision | □ | □ | □ | □ |
| Increased sensitivity to light | □ | □ | □ | □ |
| Colored halos around objects | □ | □ | □ | □ |
| Feeling that sight is worsening | □ | □ | □ | □ |
| Headache | □ | □ | □ | □ |
