# Supplementary material for: Contribution of Total Screen/Online-Course Time to Asthenopia in Children During COVID-19 Pandemic via Influencing Psychological Stress
Source: Front Public Health. 2021 Dec 1;9:736617. doi: 10.3389/fpubh.2021.736617 (PMC8671164; doi:10.3389/fpubh.2021.736617)
Supplement: Supplementary file 6 [file Data_Sheet_3.DOCX]

**Supplementary Method 2.** Definition of asthenopia and re-validation of the Chinese version of CVS-Q using the item response theory

**Definition of asthenopia**

The computer vision syndrome questionnaire (CVS-Q) for determining visual fatigue (1), ^2^was translated into Chinese to make it more in line with the Chinese thinking pattern. For the measurement of asthenopia, all participants were asked about the frequency (never, occasionally, often, or always) and the intensity (never, mild, moderate, or intense) of each of the following 16 symptoms: burning, itching, feeling of a foreign body, tearing, excessive blinking, eye redness, eye pain, heavy eyelids, dryness, blurred vision, double vision, difficulty focusing for near vision, increased sensitivity to light, colored halos around objects, feeling worsening eyesight, and headache. Then asthenopia was defined by the following steps:

Firstly, the score of each item was defined as the multiplication of its frequency and intensity of each item, i.e.,

$${Score}_{i}={Frequency}_{i}\times{Intensity}_{i}, i=1, 2,\ldots,16$$

where “never”, “occasionally”, and “often or always” were coded as 0, 1, and 2, respectively, for frequency; and “never or mild”, “moderate”, and “intense” were coded as 0, 1, and 2, respectively, for intensity. Then, each ${Score}_{i}$ would have four possible values, i.e., 0, 1, 2, or 4.

Secondly, ${Newscore}_{i}$ $\left( i=1, 2,\ldots,16 \right)$was defined by collapsing the values for each of ${Score}_{i}$, i.e., 0 was kept as 0, 1 or 2 was re-coded as 1, and 4 as 2.

Thirdly, the total score was defined as the sum of all the scores:

$$Total score=\sum_{i=1}^{16} {Newscore}_{i}$$

Finally, if the total score was ≥ 6, then the student was considered to suffer asthenopia.

**Re-validation of Chinese version of CVS-Q**

We re-validated the translated Chinese version of CVS-Q using the item response theory (IRT) in a pilot study comprising 516 high school tenth and eleventh graders (2). The mean age of the participants was 16.6 [standard deviation (SD)=0.8] years old. Slightly more than half of the students are girls (*n*=271, 52.5%). The total score for 16 items ranges from 0 to 32. Their scores on the CVS-Q subscales are presented in Supplementary Method 2. Table 1. Girls had higher total severity scores and scores in four subscales, i.e., “eye pain,” “heavy eyelids,” “dryness,” and “feeling that sight is worsening.”

For each item of the 16 symptoms [i.e.,${Newscore}_{i}$ $\left( i=1, 2,\ldots,16 \right)$], the graded response models (GRM), basically an ordered logistic model, was selected from four candidate models, i.e., partial credit model (PCM), generalized PCM, rating scale model (RCM), and GRM, by using Akaike’s and Schwarz’s Bayesian information criteria (AIC and BIC). GRM was used to predict the cumulative probability of responding in a particular item response category of higher as a function of the individual’s underlying (latent) asthenopia symptom levels [i.e., an IRT score ($\theta$)], item difficulty level parameters for the kth category ($b_{ik}$), and an item discrimination parameter ($a_{i}$). The results (Supplementary Method 2. Table 2) showed that the discrimination parameters ranged from 2.70 (95% CI 2.15 to 3.15) (for item “feeling that sight is worsening”) to 5.91 (95% CI 4.32 to 7.50) (for item “difficulty focusing for near version”). The item with the highest difficulty (location) is “colored halos around objects.” The students located at 1.23 on the $\theta$ scale has the same probability of being scored as 0 as to 1 or higher, and the students with an $\theta$ of 2.18 have a probability of 50% to have a severity score of 2, and the same probability of having a severity score of 0, and 1, which was further highlighted in the boundary characteristic curves (BCCs) [Supplementary Method 2. Figure 1-(I)]. For each item, the same discrimination parameter applies for the successive dichotomizations (i.e., ≥ 1 vs. < 1, and ≥ 2 vs. < 2). The different discrimination parameters across items determine different steepness in BCCs’ center part. The category characteristic curves (CCCs) are presented in Supplementary Method 2. Figure 1-(II). Listing the item “headache” as an example, the students with an $\theta$ approximately lower than 0.9 are most likely to respond in the lowest category (i.e., with a severity score is 0). Similarly, those with an $\theta$ approximately greater than 1.9 are most likely to respond in the highest category (i.e., with a severity score of 2); and those in between are most likely to have a severity score of 1.

The test information function for the analyzed set of 16 items as an overall asthenopia, and its standard error are reported in Supplementary Method 2. Figure 2-(I). The scale approximately between 0.8 and 2.2 offers the highest information. This figure suggests that the scale consists of 16 items under consideration could be used as a tool evaluating with high precision for most of the population, excluding the people with low or very high severity of CVS. For individuals located at $\theta$=0.95, 1.42, or 1.92 and high, the expected score is greater than 8, 16, and 24, respectively [Supplementary Method 2. Figure 2-(II)].**Supplementary Method 2. Table 1** Characteristics of participants by sex, a pilot study

| **Characteristics** | **Total** | **Boys** | **Girls** | ***p* Value^a^** |
| --- | --- | --- | --- | --- |
| No. of participants (%) | 516 (100) | 245 (47.5) | 271 (52.5) | NA |
| Age, mean (SD) | 16.57 (0.77) | 16.61 (0.78) | 16.54 (0.76) | 0.291 |
| Score in CVS-Q^b^ |  |  |  |  |
| 1 Burning | 423/84/9 | 203/39/3 | 220/45/6 | 0.591 |
| 2 Itching | 380/119/17 | 186/54/5 | 194/65/12 | 0.218 |
| 3 Feeling of a foreign body | 428/80/8 | 205/38/2 | 223/42/6 | .630 |
| 4 Tearing | 389/107/20 | 183/54/8 | 206/53/12 | 0.797 |
| 5 Excessive blinking | 414/83/19 | 197/38/10 | 217/45/9 | 0.961 |
| 6 Eye redness | 415/85/16 | 194/41/10 | 221/44/6 | 0.449 |
| 7 Eye pain | 390/104/22 | 199/40/6 | 191/64/16 | 0.004 |
| 8 Heavy eyelids | 393/97/26 | 198/35/12 | 195/62/14 | 0.025 |
| 9 Dryness | 390/100/26 | 197/38/10 | 193/62/16 | 0.016 |
| 10 Blurred vision | 394/106/16 | 195/42/8 | 199/64/8 | 0.117 |
| 11 Double vision | 432/73/11 | 205/32/8 | 227/41/3 | 0.893 |
| 12 Difficulty focusing for near vision | 434/72/10 | 205/33/7 | 229/39/3 | 0.736 |
| 13 Increased sensitivity to light | 393/101/22 | 191/44/10 | 202/57/12 | 0.375 |
| 14 Colored halos around objects | 449/61/6 | 212/28/5 | 237/33/1 | .704 |
| 15 Feeling that sight is worsening | 366/116/34 | 192/43/10 | 174/73/74 | <0.001 |
| 16 Headache | 392/103/21 | 195/41/9 | 197/62/12 | 0.073 |
| Total score, median (25^th^-75^th^ percentile) | 0 (0-6) | 0 (0-5) | 1 (0-7) | 0.002 |

Abbreviations: COVID, coronavirus disease; CVS-Q, computer vision syndrome-questionnaire; NA, not applicable; SD, standard deviation.

^a^ *p* values for the difference in related variables between boys and girls were calculated from Student’s t test for independent two samples (age), or Wilcoxon rank-sum test (CVS-Q related items).

^b^ Data are counts in the three categories (i.e., 0, 1, and 2) for each item, if not specified.

**Supplementary Method 2. Table 2** Item parameter estimates for calibrated CVS-Q IRT model in the pilot study

| **CVS-Q items** | **Item discrimination**  **parameter [**$\boldsymbol{a}_{\boldsymbol{i}}\boldsymbol{(95\%}\mathbf{CI}$**)]** | **Item difficulty**  **parameter [**$\boldsymbol{b}_{\boldsymbol{ik}}\boldsymbol{(95\%}\mathbf{CI}$**)]** | |
| --- | --- | --- | --- |
|  |  | $\boldsymbol{b}_{\boldsymbol{i}\boldsymbol{1}}\boldsymbol{(95\%}\mathbf{CI}$**)** | $\boldsymbol{b}_{\boldsymbol{i}\boldsymbol{2}}\boldsymbol{(95\%}\mathbf{CI}$**)** |
| 1 Burning | 4.35 (3.32, 5.38) | 1.06 (0.94, 1.18) | 2.11 (1.82, 2.39) |
| 2 Itching | 3.22 (2.53, 3.90) | 0.82 (0.70, 0.93) | 2.03 (1.76, 2.30) |
| 3 Feeling of a foreign body | 5.36 (3.99, 6.73) | 1.08 (0.96, 1.19) | 2.07 (1.80, 2.35) |
| 4 Tearing | 3.27 (2.57, 3.97) | 0.87 (0.75, 0.99) | 1.95 (1.69, 2.20) |
| 5 Excessive blinking | 4.20 (3.24, 5.17) | 1.02 (0.90, 1.13) | 1.83 (1.61, 2.06) |
| 6 Eye redness | 4.44 (3.39, 5.49) | 1.00 (0.89, 1.11) | 1.88 (1.65, 2.11) |
| 7 Eye pain | 5.27 (4.04, 6.50) | 0.84 (0.74, 0.94) | 1.71 (1.52, 1.90) |
| 8 Heavy eyelids | 4.81 (3.72, 5.89) | 0.87 (0.76, 0.97) | 1.68 (1.49, 1.86) |
| 9 Dryness | 4.35 (3.40, 5.30) | 0.85 (0.75, 0.96) | 1.70 (1.51, 1.90) |
| 10 Blurred vision | 4.42 (3.42, 5.42) | 0.87 (0.76, 0.98) | 1.88 (1.65, 2.12) |
| 11 Double vision | 5.51 (4.08, 6.94) | 1.11 (0.99, 1.22) | 1.94 (1.70, 2.19) |
| 12 Difficulty focusing for near vision | 5.91 (4.32, 7.50) | 1.11 (1.00, 1.23) | 1.95 (1.70, 2.19) |
| 13 Increased sensitivity to light | 3.87 (3.03, 4.70) | 0.89 (0.78, 1.00) | 1.82 (1.59, 2.04) |
| 14 Colored halos around objects | 5.14 (3.75, 6.52) | 1.23 (1.11, 1.36) | 2.18 (1.86, 2.49) |
| 15 Feeling that sight is worsening | 2.70 (2.15, 3.25) | 0.77 (0.65, 0.89) | 1.81 (1.57, 2.04) |
| 16 Headache | 3.28 (2.59, 3.98) | 0.90 (0.78, 1.02) | 1.90 (1.65, 2.15) |

Abbreviations: CI, confidence interval; CVS-Q, computer vision syndrome-questionnaire; IRT, item response theory.

The graded response models (GRM) were used for the analysis. The rating scale for each item had three categories: 0, 1, and 2.

**Supplementary Method 2. Figure 1**

**Pr(item≥2)**

**Pr(item≥1)**

**(I)**

**(II)**

**Pr(item=1)**

**Pr(item=0)**

**Pr(item=2)**

The boundary characteristic curves [BCCs, (I)], and category characteristic curves (CCCs, (II)] for all sixteen severity scores of CVS-Q. The graded response models (GRM) were used for the analysis. The rating scale for each item had three categories: 0, 1, and 2.

**Supplementary Method 2. Figure 2**

**(I)**

**(II)**

The test information function and standard error (I), and the test characteristic curves with expected total score as 8, 16 and 24 (II) for the set of sixteen items used. The graded response models (GRM) were used for the analysis. The rating scale for each item had three categories: 0, 1, and 2.

**REFERENCES**

1. Segui Mdel M, Cabrero-Garcia J, Crespo A, Verdu J, Ronda E. A reliable and valid questionnaire was developed to measure computer vision syndrome at the workplace. J Clin Epidemiol. (2015). 68(6):662-73.

2. StataCorp. Stata item response theory reference manual (release 16). College Station, TX: Stata Press; 2019.
